# Investigation of and Response to Autochthonous Dengue, Los Angeles County, California, USA, August–November 2024

**DOI:** 10.3201/eid3205.251812

**Published:** 2026-05

**Authors:** Aisling M. Vaughan, Claire Park, Van P. Ngo, Zuelma A. Contreras, Jordan John Lee, Phoebe Danza, Meredith Haddix, Olivia Moir, Nicole Green, Michael Brown, Taylor Burleson, Amy Marutani, Ashley Nicholas, Tristan Hallum, Steve Vetrone, Liza Ortiz, Gladys Fernandez, Eric El-Tobgy, Jose Escobar, Tricia-Nicole Gandela, Cristin Mondy, Jan King, Brandon Dean, Elizabeth Rubin, Pablo Valadez, Stella Fogleman, Dawn Terashita, Sharon Balter, Umme-Aiman Halai

**Affiliations:** Epidemic Intelligence Service, Centers for Disease Control and Prevention, Atlanta, Georgia, USA (A.M. Vaughan); Los Angeles County Department of Public Health, Los Angeles, California, USA (A.M. Vaughan, C. Park, V.P. Ngo, Z.A. Contreras, J.J. Lee, P. Danza, M. Haddix, O. Moir, L. Ortiz, G. Fernandez, E. El-Tobgy, J. Escobar, T.-N. Gandela, C. Mondy, J. King, B. Dean, E. Rubin, P. Valadez, S. Fogleman, D. Terashita, S. Balter, U.-A. Halai); Public Health Laboratory, Los Angeles (N. Green, M. Brown, T. Burleson, A. Marutani, A. Nicholas); San Gabriel Valley Mosquito and Vector Control District, Los Angeles (T. Hallum); Greater Los Angeles County Vector Control District, Los Angeles (S. Vetrone)

**Keywords:** dengue, dengue fever, dengue virus, viruses, vector-borne infections, mosquitoborne virus, autochthonous, outbreak, California, United States

## Abstract

Dengue is not endemic in the continental United States; most cases occur in returning travelers. During August–November 2024, a total of 14 locally acquired cases of dengue were identified in Los Angeles County, California, USA. Epidemiologic evidence indicates that locally acquired cases occurred in several neighborhoods, suggesting short transmission chains after introductions from returning travelers. In one neighborhood, evidence supported ongoing transmission for up to 7 weeks. Median patient age was 54 (range 5–79) years; 8 (57%) patients were female and 6 (43%) male, and 6 (43%) required hospitalization. Delays in healthcare seeking and diagnoses were noted; median time from symptom onset to specimen collection for dengue testing was 9 (range 2–34) days. Local dengue transmission in Los Angeles County highlights the emerging threat of mosquitoborne disease transmission in nonendemic areas and the need for rapid and coordinated public health and vector control responses to interrupt transmission.

Dengue, a mosquitoborne disease caused by 4 dengue virus types (DENV-1–4), is transmitted mainly by *Aedes aegypti* and *Ae. albopictus* mosquitoes. Symptoms usually appear 2–7 days after infection and can include fever, headache, retro-orbital pain, joint and muscle pain, and rash. Most cases are mild, but severe dengue can cause bleeding, shock, and death. Dengue causes substantial global illness and death. In 2024, global incidence reached record levels (*1*); ≈13 million cases were reported in the Americas, far surpassing previous years (*2*). Dengue is not endemic in the continental United States; most cases occur in returning travelers, but sporadic local transmission has been documented, including in California (*3*).

Dengue is reportable in Los Angeles County (LAC), California, where a marked increase in travel-associated cases has been observed. In 2024, a total of 222 travel-associated cases were reported in the LAC Department of Public Health (LACDPH) jurisdiction, compared with 35 during 2022 and 75 during 2023, closely mirroring global increases in dengue activity. In addition, sporadic locally acquired dengue cases were reported by the Pasadena and Long Beach Public Health Departments in LAC in 2023; however, no evidence of sustained transmission was identified (*4*,*5*).

On August 30, 2024, LACDPH was notified of a positive dengue laboratory result for a person with no recent travel history. Subsequently, several additional autochthonous dengue cases were identified across LAC. We describe the public health and vector control measures implemented during August–November 2024 to rapidly identify cases and prevent further local transmission.

## Methods

### Case and Outbreak Identification

Healthcare providers or laboratories are mandated to report dengue cases to LACDPH, which coordinates laboratory confirmation; interviews patients to collect symptom onset, type and duration of symptoms, hospitalization, exposure and travel history; and reviews household composition and medical records. Cases are classified according to the Council of State and Territorial Epidemiologists definition (*6*). Locally acquired (autochthonous) dengue refers to infections acquired through local mosquito transmission, as distinguished from travel-associated cases, in which infections are acquired elsewhere. A case is classified as locally acquired if the patient had no travel to dengue-risk areas during the 2 weeks before symptom onset. An outbreak was considered when >2 locally acquired cases occurred in patients with symptom onset dates 2–8 weeks apart and primary exposure sites located <1 mile apart, suggesting sustained local transmission. That timeframe accounts for the combined human incubation period (5–7 days) and mosquito extrinsic incubation period (8–12 days); the 1-mile radius reflects the limited flight range of *Aedes* mosquitoes (≈150 meters).

### Public Health Response

LACDPH had initiated an intradepartmental planning effort between principals in the Disease Control and Health Protection bureaus in anticipation of a local outbreak, using Centers for Disease Control and Prevention (CDC) Public Health Emergency Preparedness funding to develop, train, and exercise response plans. CDC guidance (*7*) was also used to inform the Dengue Response Plan. 

Response operations targeted a 150-meter radius around the patient residence (i.e., the flight span of an *Aedes* mosquito). A field command post was established to coordinate the deployment of LACDPH staff, including providing just-in-time training, safety instructions, and logistical support such as water, mosquito repellant, and radio communications. An overview of the outreach population was produced by linking census-level data from the 2002 Population Estimates Program (*8*) with parcels and addresses from the 2023 County Tax Assessors Parcels dataset and census tract–level demographic characteristics from the 2020 American Community Survey. Metrics included the number of residential and nonresidential buildings, primary languages spoken, race, ethnicity, age distribution, and household size. New cases identified from enhanced surveillance expanded the operational fields. Enhanced surveillance included offering testing to all household members, attempts to contact nearby households, and reviewing emergency department visit data in the affected area (Appendix).

### Vector Control Response

Five independent vector control districts (VCDs) serve the LACDPH jurisdiction. When a locally acquired dengue case is identified, LACDPH notifies the relevant VCD, which implements enhanced, targeted mosquito control activities within the 150-meter radius around the patient’s residence. Activities include door-to-door property inspections, education, increased mosquito trapping and testing, and larvicidal and adulticidal mosquito abatement.

### Laboratory Testing

Healthcare providers and laboratories report positive results to LACDPH. Specimens were sent to the public health laboratory (PHL) for confirmatory PCR and nonstructural protein (NS) 1 testing, following CDC guidelines (*9*). If PCR and NS1 results were negative or PCR serotyping was not available, samples were referred to the California Department of Public Health’s Viral and Rickettsial Disease Laboratory (VRDL) for plaque reduction neutralization testing (PRNT) or to CDC for PRNT and serotyping.

Samples from patients with compatible symptoms and equivocal DENV-specific IgM or positive IgG results also underwent additional IgM and PCR testing, per CDC guidance. In addition, because of the potential for cross-reactivity with other flaviviruses, febrile patients without neuroinvasive disease who tested positive for West Nile virus (WNV) were also tested for dengue by IgM, PCR, and NS1. Household members and neighbors of confirmed patients were tested by dengue NS1 and IgM assays, and PCR was performed on positive specimens.

## Results

### Initial Case Detection

On August 30, 2024, LACDPH was notified of a positive IgM dengue laboratory result in a resident of neighborhood A, located in the central San Gabriel Valley region of LAC (patient 1) (Figure). The patient, who sought emergency department care in mid-August, had fever, leukopenia, and thrombocytopenia and tested positive for dengue IgM. Medical record review and patient interview confirmed no recent travel. LACDPH notified the San Gabriel Valley Mosquito and Vector Control District, which initiated mosquito control activities within a 150-meter radius of the patient’s residence. PHL performed additional testing for dengue NS1 antigen and PCR; both results were positive, confirming DENV-3 infection. Confirmatory PRNT testing performed at the California Department of Public Health’s VRDL also yielded a positive result.

**Figure:**
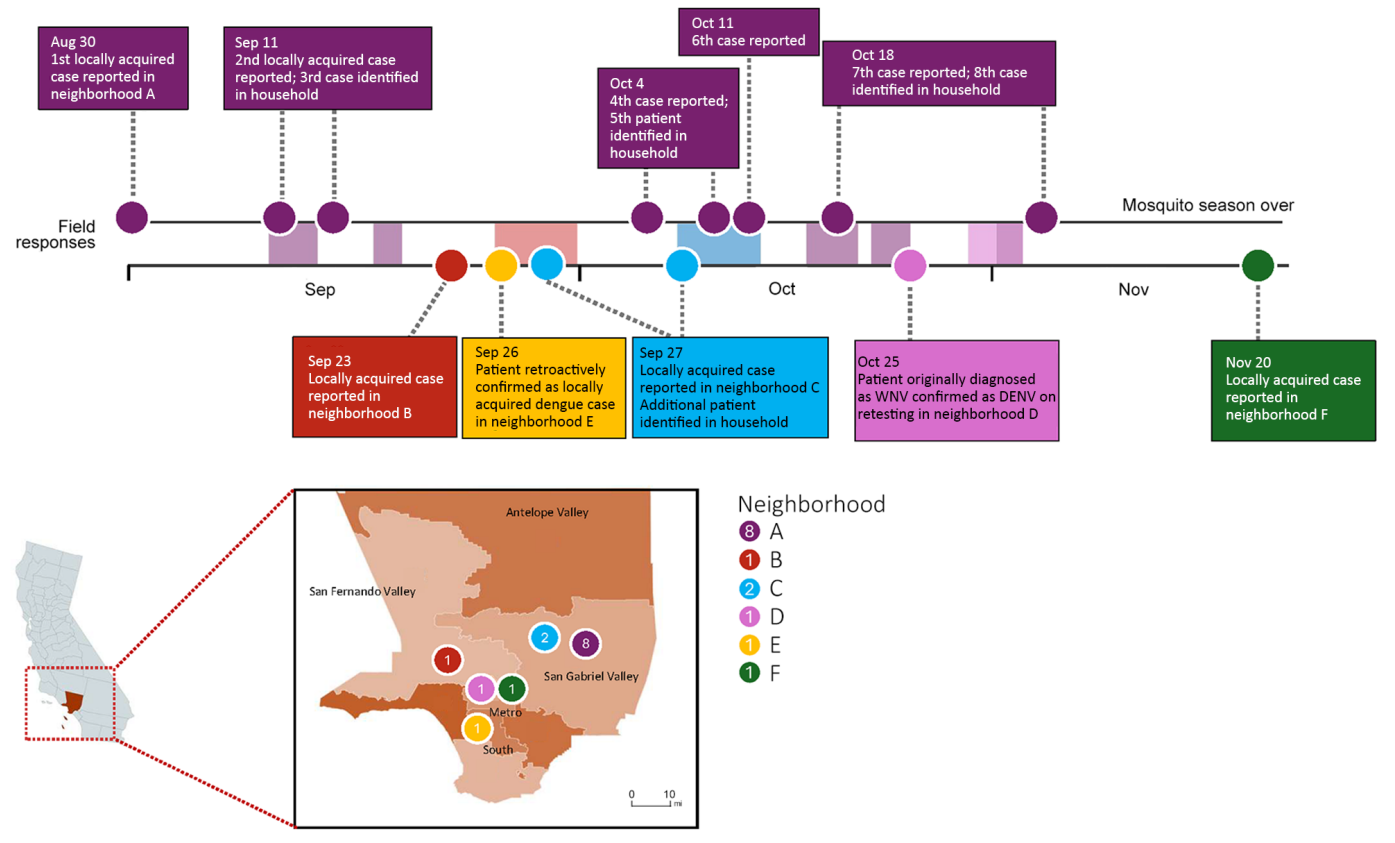
Timeline and geographic distribution of autochthonous dengue cases and public health response, Los Angeles County, California, USA, 2024. A) Timeline of cases (circles) by date of report, or date of confirmation (positive laboratory result) for a household member. Colors indicate residential neighborhood (see key in panel B); shaded bars indicate vector control and public health response activities in each neighborhood after identification of a locally acquired case. B) Map of Los Angeles County. Circles indicate cases identified by neighborhood; numbers indicate total cases per neighborhood. Case identified in neighborhood E was initially classified as a travel-related case; however, retrospective review revealed travel only to areas without ongoing DENV transmission. Based on that information, the case was reclassified as locally acquired. Inset shows location of Los Angeles County within California. DENV, dengue virus; WNV, West Nile virus.

### Outbreak Detection

Active case finding was launched within a 150-meter radius of the patient’s residence using an established response plan. All household members and neighbors reached within the radius were offered testing and provided guidance on dengue prevention, mosquito breeding reduction, and healthcare seeking. During the field response, a healthcare provider reported a case in a person residing near the first patient (patient 2). In response, testing was offered to household members, and the outreach radius was expanded to incorporate households within 150 meters of the new case; the household testing identified a third case (patient 3). Shortly thereafter, another case was reported in a person who lived <1 mile from the initial cluster (patient 4); testing of household members identified a fifth case in a child (patient 5). Patients 4 and 5 attended a place of worship with an adjoining school, identified as a potential secondary exposure site; we conducted outreach and testing at that location. A sixth case (patient 6), reported to LACDPH by a laboratory, lived near patient 1 outside the 150-meter response area and was not identified during the field response. All 6 cases were identified within a 1-mile radius over a 7-week period, consistent with an outbreak involving ongoing local transmission. Finally, another case (patient 7) was reported by a laboratory after an 11-day hospital stay during which dengue was not suspected. Subsequent household testing identified an additional case (patient 8). Those cases were farther from the original cluster (3 miles) but closer to the place of worship and school (1.7 miles). Overall, 8 locally acquired dengue cases were identified in neighborhood A.

### Additional Introductions

During September–November, 6 additional dengue cases were identified across 5 distinct areas in LAC; none had relevant travel history. The first patient, reported to LACDPH by a healthcare provider, resided in neighborhood B, ≈37 miles from neighborhood A in the San Fernando Valley. During the field response, 2 workplaces were identified within the operation radius, and LACDPH deployed a mobile response unit to offer testing to all employees; no additional cases were identified. The second patient resided in neighborhood C, 5 miles from neighborhood A in the San Gabriel Valley, and sought testing for dengue after multiple unsuccessful attempts at a diagnosis; a laboratory notified LACDPH of that case. A symptomatic household member also tested positive for DENV, but no further cases were identified in the surrounding areas. Additional patients were identified in multiple different areas >20 miles from neighborhood A. The fourth patient, in neighborhood D in the city of Los Angeles, was initially misdiagnosed with WNV; however, subsequent testing by PCR and PRNT requested by LACDPH confirmed DENV infection. Public health and vector control activities were promptly implemented in response to those cases to mitigate further spread. Finally, 2 patients were identified in neighborhood E in Los Angeles and neighborhood F in South Los Angeles and reported to LACDPH by laboratories. However, because of low mosquito activity at that time of year, no door-to-door field responses were mounted in response to those cases.

### Enhanced Surveillance

In total, 9 field responses were conducted in LAC in response to case detections for locally acquired dengue. Public health responses were extensive, requiring 16 field operations (1 field operation per day); 866 households, 2 worksites, and 1 place of worship and school were reached during the emergency response, resulting in 318 completed household surveys. Overall, 245 (28%) households declined participation, and 303 (35%) households could not be reached (reasons included properties were determined as unsafe to access, marked as vacant after 2 visits, or no response was received during outreach).

A total of 224 persons were tested for dengue during the response; 2 were IgM-positive for DENV. One sample from an IgM-positive person also had confirmatory PRNT testing performed at CDC, which was positive for DENV-1; that person did not report symptoms and did not meet the case definition. The second IgM-positive case-patient was also positive for NS1 and reported compatible symptoms but had traveled recently to an area with ongoing dengue transmission. Therefore, neither patient met the criteria for locally acquired dengue.

Of 314 households surveyed, 44% reported that household members had experienced mosquito bites during the previous 2 months. Twenty-three (8%) of 301 households reported that a resident was symptomatic; however, only 1 of those noted as symptomatic was successfully reached for testing during field responses. One in 5 households (18%; 57/313) reported that a household member had traveled internationally or out of state during the previous 2 months. Many households were taking measures to prevent mosquitoes on their properties and mosquito bites: eliminating standing water on the property (59%; 188/318), wearing mosquito repellent (44%; 140/318), checking screens and windows for holes or tears (43%; 138/318), and removing mosquito resting sites (40%; 126/318). However, 11% (34/318) households reported that they do not do anything to prevent mosquitoes. At the time of the survey, 46% (143/314) of households reported that they were already aware of a local case of dengue in LAC, primarily from the news (45%; 65/143), a notice from LACDPH (27%; 39/143), or social media (13%; 18/143).

Syndromic surveillance for dengue in LAC was conducted during September–December, continuing for >45 days after the last reported locally acquired case. During that period, 81 dengue-compatible detections were identified across 4 postal (ZIP) codes and surrounding areas. After review, LACDPH requested medical records for 12 patients who exhibited compatible clinical symptoms without an alternative diagnosis. Specimens were unavailable for 11 (92%) of those patients, and medical record review did not indicate sufficient clinical suspicion of dengue to warrant new specimen collection. One patient’s specimen was tested at PHL and VRDL, with negative results for DENV. No additional locally acquired dengue cases were identified through syndromic surveillance.

### Patient Characteristics

For 14 locally acquired dengue cases, median patient age was 54 (range 5–79) years; 8 (57%) patients were female and 6 (43%) male (Table). Four (29%) cases were identified through patient household testing by public health; the remainder were detected through healthcare providers. Eight (57%) patients, including 3 household cases, lived in the same neighborhood (neighborhood A); 6 (43%) of those resided within 1 mile of another patient.

**Table:** Characteristics of patients with locally acquired dengue cases, Los Angeles County, California, USA, August–November 2024*

Characteristic	Value
Total	14 (100)
Median age, y (range)	54 (5–79)
Age group, y	
0–19	1 (7)
20–39	2 (14)
40–59	8 (57)
>60	3 (21)
Sex	
F	8 (57)
M	6 (43)
Location	
Neighborhood A	8 (57)
Neighborhood B	2 (14)
Neighborhood C	1 (7)
Neighborhood D	1 (7)
Neighborhood E	1 (7)
Neighborhood F	1 (7)
Epidemiologic link	
Household	4 (29)
Neighborhood†	6 (43)
None	4 (29)
Mosquito exposure	
Y	6 (43)
N	4 (29)
Unknown	4 (29)
Symptoms	
Fever	14 (100)
Headache	8 (57)
Nausea and vomiting	8 (57)
Chills	7 (50)
Rash	6 (43)
Muscle pain	5 (36)
Joint pain	4 (29)
Diarrhea	2 (14)
Eye pain	1 (7)
Hospitalization	
Y	6 (43)
N	8 (57)
Serotype	
DENV-3	8 (57)
DENV-1 or −3‡	2 (14)
DENV-1	1 (7)
Unknown	3 (21)

*Values are no. (%) except as indicated. Percentages are rounded to the nearest whole number and may not sum to 100%. DENV, dengue virus. †Resided within 1 mile of a case (includes index patient; includes 2 patients identified through household testing). ‡Indeterminant.

Six (42.9%) patients reported known mosquito exposure, whereas 4 (28.6%) patients reported no history of mosquito bites before symptom onset. All patients reported fever; 8 (57%) had headache, and 8 (57%) had nausea and vomiting. Six (43%) patients required hospitalization. No patients met criteria for severe dengue or dengue hemorrhagic fever, and none died. Infection history was unknown for all patients.

All 14 patients were tested for DENV IgM; 12 were positive, 1 was equivocal, and 1 was negative. PHL performed confirmatory testing on all specimens using reverse transcription PCR and NS1 testing. Nine (64%) patients tested positive by NS1 testing, reverse transcription PCR, or both. Additional confirmatory testing using PRNT was performed for 8 (57%) patients, 5 whose specimens were negative by both PCR and NS1 testing and 3 who had positive PCR or NS1 testing but no available serotype information. DENV serotypes were available for 11 (79%) patients; DENV-3 was confirmed for 8, DENV-1 for 1, and indeterminant for 2 (DENV-1 or DENV-3). Sequencing results were available for only 1 patient and confirmed DENV-3 infection.

Median time from symptom onset to seeking medical care was 2 (range 1–14) days. The median interval from healthcare encounter to specimen collection was 3.5 (range 0–27) days. Overall, median time from symptom onset to specimen collection was 10 (range 4–34) days, and median interval from specimen collection to reporting to LACDPH was 5 (range 1–17) days. Once a case was reported to LACDPH, most case interviews were completed <2 days after notification. Four (31%) cases were first identified through public health investigation rather than clinical diagnosis. All 4 patients reported symptoms compatible with dengue; 2 had previously sought medical care but were not tested for dengue at that time, and 1 was identified through retrospective testing of residual clinical specimens.

### Vector Control Response

Adult mosquito surveillance using traps placed within a 150-meter radius of case residences collected a mixture of *Culex*, *Aedes*, and *Culiseta* spp. mosquitoes; *Cx. quinquefasciatus* mosquitoes were most abundant. Among *Aedes* mosquitoes, *Ae. aegypti* was the dominant species detected near case households; *Ae. albopictus* was detected in low abundance. During property inspections, breeding sources near the case households were documented; however, formal larval indices were not calculated because they are not considered informative for risk assessment (*10*). Female *Ae. aegypti* mosquitoes collected at each trapping site within the response areas were tested for DENV, chikungunya virus, and Zika virus; however, no mosquitoes tested positive for DENV.

### Public and Healthcare Provider Outreach

To raise public awareness and promote dengue prevention, LACDPH implemented messaging campaigns focused on persons who work or reside within a 150-meter radius of confirmed locally acquired cases. Educational materials were made available in multiple languages on the basis of population needs to ensure broad accessibility. During the response, LACDPH issued 6 press releases and held a press conference in neighborhood A to enhance public awareness and deliver timely risk communication. A dedicated dengue webpage provided real-time updates and served as a central resource for prevention and control resources for the public. LACDPH issued 2 health advisories to alert healthcare providers about the risks of local DENV transmission in LAC, emphasizing the need to consider dengue in patients with consistent symptoms, regardless of travel history, in the absence of an alternative diagnosis; order appropriate diagnostic testing for suspected dengue infection; counsel patients and travelers on prevention strategies; and promptly report dengue cases to LACDPH to enable timely public health response (*11*).

## Discussion

In 2024, LACDPH identified 14 locally acquired dengue cases in LAC, likely from multiple introductions by infected returning travelers. Epidemiologic evidence suggests that >1 introduction resulted in sustained local transmission, demonstrating the potential for dengue to emerge in nonendemic areas of the United States that have competent *Aedes* mosquito vectors and suitable environmental conditions. In response, LACDPH and partners rapidly implemented intensive public health and vector control measures, including active case finding, mosquito control, and community outreach around patient residences, to mitigate spread, raise awareness, and promote prevention. The ability to mobilize quickly and coordinate across sectors was aided by the existing LAC Dengue Response Plan, which provided a structured multisectoral response framework and established communication channels to coordinate efforts across VCDs, laboratories, and state and federal partners. In addition to dengue preparedness planning, LAC maintains response plans for other arboviruses, including Zika virus; a chikungunya response plan is under development.

Vector control was central to mitigating DENV spread in areas at risk for local transmission. Unlike many other jurisdictions, LAC is served by multiple independent VCDs, each with its own governance, operational protocols, and resource levels. This decentralized system means that vector surveillance, outbreak response, and mosquito control activities depended on the capacity and expertise of the individual districts. During this investigation, local vector control districts identified established *Ae. aegypti* mosquito populations near case households; however, no DENV-positive mosquitoes were detected. The absence of positive mosquito pools during localized outbreaks should be interpreted with caution because mosquito infection prevalence can be very low. Studies from Puerto Rico reported very low mosquito infection rates even during periods of active dengue transmission, indicating that the absence of DENV-positive mosquito pools does not exclude ongoing transmission (*12*,*13*).

During this outbreak, enhanced surveillance played a crucial role in identifying dengue infections that might otherwise have gone undetected, improving understanding of the extent of local transmission and enabling more tailored public health responses to interrupt transmission. Of the 6 dengue infections identified, 4 were household members of previously confirmed patients, highlighting the importance of household testing. Dengue virus infections often cluster within households and nearby areas, making household testing an effective strategy to identify cases and interrupt transmission chains. However, neighborhood-level door-to-door testing, although valuable for understanding the extent of local transmission, was resource-intensive and required substantial staffing and logistical coordination, making it unsustainable in the long term. Overall participation rates in door-to-door outreach were modest because of declined participation or lack of reach (vacancy or nonresponse), which might have resulted in underascertainment of cases and limited our ability to fully characterize the extent of local transmission. Although persons reported symptoms during neighborhood outreach, testing proved challenging because many declined or were unavailable during the field response and did not follow up with their healthcare provider or attend mobile health clinics. Workplace testing was also challenging, in part because of lack of communication with staff and barriers to scheduling during work hours. In contrast, a community testing event at a place of worship and school, organized in collaboration with trusted community partners, achieved a high turnout; 68 persons were tested for dengue. Future responses should prioritize more resource-efficient strategies, including patient household testing and community events, while enhancing provider and public awareness.

Another limitation of this investigation is that surveillance relied primarily on identifying symptomatic cases consistent with the Council of State and Territorial Epidemiologists dengue case definition. Because dengue infections are frequently asymptomatic or mildly symptomatic, a substantial number might have gone undetected. As a result, the number of infections identified during this investigation might underestimate the true extent of transmission. Future serologic studies could clarify the extent of transmission and inform prevention and control activities.

For this outbreak, health information was disseminated via press releases, social media campaigns, press conferences, and in-person outreach during field activities. Messaging was delivered in multiple languages to ensure accessibility across diverse communities. Those efforts informed residents about dengue risk and encouraged measures to reduce mosquito breeding and avoid bites. Going forward, proactive risk communication will be delivered ahead of the mosquito season in LAC, aiming to raise awareness of vectorborne disease risk and provide the public with tools to prevent infection.

Despite efforts to raise awareness among healthcare providers, clinical recognition of locally acquired dengue remained limited, and several cases were not initially suspected as dengue. That challenge is heightened in nonendemic settings because symptoms during the acute phase overlap with those of other viral illnesses, and clinical suspicion is often low in the absence of travel history. That issue was evident in the delays in diagnosis of the local cases. In this investigation, the median time from symptom onset to specimen collection was 10 (range 4–34) days, often exceeding the optimal diagnostic window for PCR and NS1 detection during the first week of illness (*9*), and PRNT confirmatory testing was required for several patients. Those delays likely reflected both delayed healthcare seeking and missed opportunities for DENV testing during clinical encounters, highlighting the need for increased awareness of locally acquired dengue among the public and clinicians. Given the potential for dengue to emerge in nonendemic areas, clinicians should maintain a high index of suspicion for dengue in febrile patients with compatible symptoms, regardless of travel history, and prioritize timely testing to aid in early detection, prevent further transmission, and ensure prompt recognition and management of severe disease.

In addition to clinical challenges, laboratory testing practices contributed to diagnostic delays. In several cases, CDC-recommended testing (*9*) was not consistently performed by providers, requiring follow-up confirmatory testing at PHL and state or federal laboratories, extending the time to confirm diagnosis. Once testing was performed, reporting and public health response occurred relatively rapidly. Delayed specimen collection and low viral loads also limited the availability of specimens suitable for genomic sequencing, restricting ability to assess genetic relationships between DENV genomes and determine whether cases represented localized spread or independent introductions. Despite those limitations, the spatial and temporal distribution of the cases suggests that multiple introductions across geographically distinct areas of LAC might have occurred during August–November 2024. Fourteen locally acquired cases were identified across 6 geographically distinct areas, separated by >5 miles and up to 37 miles, and no epidemiologic links were identified. In addition, no recent travel-associated dengue cases were reported within the postal code of the initial locally acquired cases during the outbreak period.

In conclusion, local DENV transmission occurred in LAC during a time of record global dengue incidence, active outbreaks in US territories, and ongoing travel to endemic areas (*14*,*15*), highlighting the need for heightened vigilance in nonendemic areas where *Aedes* mosquitos are established. Effective preparedness depends on coordinated partnerships and multisectoral response plans, improved clinical recognition for timely detection, and robust entomologic and epidemiologic surveillance.

Appendix Additional information for investigation of and response to autochthonous dengue, Los Angeles County, California, USA, August–November 2024.
