# Supplementary material for: Investigation of and Response to Autochthonous Dengue, Los Angeles County, California, USA, August–November 2024
Source: Emerg Infect Dis. 2026 May;32(5):720–7. doi: 10.3201/eid3205.251812 (PMC13175107; doi:10.3201/eid3205.251812)
Supplement: Appendix — Additional information for investigation of and response to autochthonous dengue, Los Angeles County, California, USA, August–November 2024. [file 25-1812-Techapp-s1.pdf]

Article DOI: https://doi.org/10.3201/eid3205.251812

EID cannot ensure accessibility for supplementary materials supplied by authors. Readers who have difficulty accessing supplementary content should contact the authors for assistance.

Investigation of and Response to Autochthonous Dengue, Los Angeles County, California, USA, August–November 2024

Appendix

Enhanced Surveillance

All household members of patients were offered dengue testing, irrespective of symptoms; testing was typically arranged during the initial patient interview. Trained clinical staff visited the patient's residence and took whole blood specimens for testing at the LAC Public Health Laboratory (PHL). Trained outreach and clinical field staff were deployed to all households within the geographic area of operation to initiate outreach, including administering surveys, collecting laboratory specimens, and providing education regarding dengue prevention. Field teams attempted multiple contacts at each household; if unsuccessful, educational materials, dengue testing information, and LACDPH contact information were shared. Neighboring household surveys within the 150-m response area collected household size, demographic characteristics, travel and symptom history (within the previous 2 months), mosquito exposure, and prevention practices. All surveys were voluntary, and consent was collected before completion. Surveys were administered using REDCap (https://project-redcap.org) on LACDPH-issued mobile devices. Field teams also used REDCap to track response activities, including the number of contact attempts and households requiring follow-up. All neighboring households were also offered free dengue testing at time of survey.

Enhanced case finding through syndromic surveillance was conducted for ≥45 days after detection of a locally acquired case. Emergency department (ED) visit data were queried for the patient residential ZIP code and adjacent areas. Records were screened for a diagnosis of dengue or fever accompanied by ≥2 other dengue-like symptoms (body ache/muscle pain, headache, nausea/vomiting, rash, or chills), excluding persons with recent travel history. Flagged records

were reviewed using information from the chief complaint, diagnosis, and triage notes and followed up if they met the criteria.
